# Early Adverse Stress and Depressive and Bipolar Disorders: A Systematic Review and Meta-Analysis of Treatment Interventions

**DOI:** 10.3389/fpsyt.2021.650706

**Published:** 2021-04-26

**Authors:** Pablo Martínez, Sergio Gloger, Dante Diez de Medina, Arantza González, María I. Carrasco, Sara Schilling, Paul A. Vöhringer

**Affiliations:** ^1^Psicomedica, Clinical and Research Group, Santiago, Chile; ^2^Departamento de Psiquiatría y Salud Mental, Hospital Clínico Universidad de Chile, Santiago, Chile; ^3^Escuela de Psicología, Facultad de Humanidades, Universidad de Santiago de Chile, Santiago, Chile; ^4^Instituto Milenio para la Investigación en Depresión y Personalidad (MIDAP), Santiago, Chile; ^5^Núcleo Milenio para Mejorar la Salud Mental de Adolescentes y Jóvenes, Imhay, Santiago, Chile; ^6^Departamento de Psiquiatría y Salud Mental Campus Oriente, Facultad de Medicina, Universidad de Chile, Santiago, Chile; ^7^Escuela de Medicina, Facultad de Medicina, Universidad de Chile, Santiago, Chile; ^8^Mood Disorders Program, Tufts Medical Center, Boston, MA, United States; ^9^Department of Psychiatry, Tufts University School of Medicine, Boston, MA, United States

**Keywords:** child abuse, bipolar and related disorders, systematic review, meta-analysis, treatment outcome and efficacy, depressive disorder

## Abstract

**Introduction:** A significant proportion of adults with depressive or bipolar disorders exposed to early adverse stressors do not adequately respond to standard treatments. This review aimed at synthesizing the evidence on the effectiveness of treatment interventions for depressive or bipolar disorders in adult individuals (aged 18 years or more) exposed to adverse stress early in life.

**Methods:** Systematic review and meta-analysis including experimental and quasi-experimental published studies indexed in CINAHL, EMBASE, PubMed, and Web of Science databases and/or in reference lists. Data management and critical appraisal (with the Study Quality Assessment Tools) was conducted independently by multiple researchers. A quality-effects model for meta-analysis was used for data synthesis and publication bias was assessed using the Doi plot and LFK index. The main outcome was short-term reductions in depressive symptoms.

**Results:** Eight randomized controlled trials, three controlled before-and-after (pre-post) studies, and three uncontrolled before-and-after studies were included. Studies lacked bipolar disorder patients. Unclear randomization procedures and reporting of blinded outcome assessor, and limited use of intention-to-treat analysis, were relevant potential sources of bias. Meta-analyses indicated that psychological, pharmacological, and combined interventions were effective in reducing depressive symptoms in the short- (Cohen's *d* = −0.55, 95% CI −0.75 to −0.36, *I*^2^ = 0%) and mid-term (Cohen's *d* = −0.66, 95% CI −1.07 to −0.25, *I*^2^ = 65.0%). However, a high risk of publication bias was detected for these outcomes. A small number of studies, with mixed results, reported interventions with long-term improvements in depressive symptomatology, and short- and mid-term response to treatment and remission.

**Conclusion:** Despite the well-documented long-lasting, negative, and costly impact of early adverse stressors on adult psychopathology, evidence on treatment alternatives remains scant. Trauma-focused treatment interventions—whether psychological interventions alone or in combination with pharmacotherapy—may have the potential to reduce the severity of depressive symptom in adults who were exposed to early adverse stress. Findings must be interpreted with considerable caution, as important study and outcome-level limitations were observed and gray literature was not considered in this systematic review and meta-analysis.

## Introduction

Depressive disorders and bipolar and related disorders are among the most challenging public mental health issues worldwide. These psychiatric conditions are leading global causes of disability (1, 2) and significant contributors to premature and excess mortality, due to suicide and associated comorbidities (3–6). For instance, recent global health estimates suggests that more than 300 million people lives with depression worldwide, that depressive disorders are the single largest contributor to years lived with disability, and that the incidence and the disability-adjusted life years for bipolar disorder have increased around fifty percent since 1990 (1, 2). Although effective psychological and drug therapies are available for these disorders, an important proportion of patients do not improve with treatment (7, 8). Notably, following a few years of recovery after treatment most of the patients will experience a relapse into depression or mania (e.g., up to 60% within 2 years) (7, 8).

Early adverse stressors—meaningful negative childhood and adolescent experiences, such as abuse and neglect, or severe household dysfunction—have been robustly linked to negative and long-lasting neurobiological and clinical consequences (9–11). Furthermore, an important body of literature has reported that early adverse stress is a risk factor for atypical, severe, chronic, and/or treatment-resistant depressive and bipolar disorders (12–24). For instance, a meta-analysis that compared the treatment response of depressed adults with or without childhood maltreatment (18) showed that depressed and maltreated individuals benefited less from standard psychological (specifically, cognitive behavioral therapy) or pharmacological treatments (e.g., tricyclic antidepressants, with mixed findings for selective serotonin reuptake inhibitors), and that particularly exhibited poorer outcomes after receiving combined interventions. Similarly, large epidemiological population-based studies and meta-analyses suggest that such traumatic experiences might be at least partly responsible for the plight of millions of adults with depressive disorders (25–28).

In light of these findings, the clinical management of depressive and bipolar disorders should consider treatment alternatives tailored to the needs of adults who were exposed to adverse stressors during childhood. However, to the best of our knowledge, no studies to date have summarized the effectiveness of treatment interventions for depressive or bipolar disorders in this subset of patients with early adverse stress. This systematic review and meta-analysis aimed at summarizing the evidence on the effectiveness of treatment interventions for depressive or bipolar disorders in adults exposed to early adverse stressors. Specifically, we look at the effect of these interventions on depressive symptoms or mania, treatment response, and clinical remission.

## Methods

This systematic review and meta-analysis was conducted in accordance with the PRISMA statement (29), using the standards outlined in the Cochrane Handbook for Systematic Reviews of Interventions (30). The study protocol was prospectively registered in PROSPERO (CRD42020165507).

### Eligibility Criteria

This systematic review included articles that were published in English or Spanish, in peer-reviewed journals, until October 2019 (with no lower limit on publication dates), indexed in CINAHL, EMBASE, PubMed, or Web of Science databases or cited in reference lists, and which had an abstract available to review.

Study eligibility criteria were defined using the PICOS model as follows:

*Population*: adult individuals (aged 18 years or more) exposed to early adverse stress before the age of 18, with a diagnosis of depressive or bipolar disorders ascertained by means of self-reported questionnaires (i.e., sample mean of symptoms above the clinical threshold for depressive or bipolar disorders) and/or clinical assessment by trained personnel.

*Intervention*: any intervention (psychological, pharmacological, psychosocial, or a combination) aimed at treating depressive or bipolar disorders in adults with early adverse stress.

*Comparator*: any intervention (psychological, pharmacological, psychosocial, or a combination) that was treatment as usual or placebo. There were also studies without a control group (note: see “*Study designs*”).

*Outcomes*: post-intervention, follow-up, and/or change in score data (i.e., change from baseline) were extracted based on self-report or clinician-rated evaluations of symptom improvement, remission, recovery, relapse and/or recurrence of depressive or bipolar disorders. To be considered in this study, outcomes must have been assessed with validated instruments or procedures. The timing of the outcomes was defined as: short-term (up to 12 weeks), mid-term (up to 24 weeks), and long-term (at least 25 weeks post-intervention).

*Study designs*: randomized controlled trials, controlled clinical trials (i.e., randomization not explicitly reported, but cannot be ruled out), interrupted time series, and before-and-after (pre-post) studies, with or without a control group.

### Search Strategy

The detailed search strategy used for each database is included in the Appendix. In general, the basic search strategy combined indexed and free-text terms for “depression” OR “bipolar” AND early adverse stress (e.g., “childhood adversities”) AND study design (e.g., “clinical trial”).

### Study Selection and Data Collection Process

After compilation of reports and removal of duplicates, the process of selecting studies (from screening reports and full-text assessment to inclusion), data coding, critical appraisal, and data extraction were carried out by four authors independently and then jointly, using previously piloted forms for study selection and data extraction. The process was documented using the PRISMA flow diagram (29). For purposes of data coding and data extraction, the following information was recorded: (1) study identification (authors, year, and country of the study); (2) characteristics of study sample and methods (including participant's age and gender, criteria used to define depressive or bipolar disorders and early adverse stress, depression symptom severity, study design, recruitment methods, and inclusion/exclusion criteria); (3) characteristics of interventions (for both arms, including type, dose, duration, frequency, and details of the contents in the case of psychological interventions or components included); (4) characteristics of study outcomes (type of outcome measure [depressive symptoms or mania, response to treatment, and remission from depressive or bipolar disorders], measurement instrument [self-report questionnaire or clinical diagnostic interview], and timing); and (5) study results (means or proportions with their standard deviations or 95% confidence intervals for continuous or dichotomous outcomes, respectively). Data were extracted on March 12th, 2020.

### Risk of Bias in Individual Studies

The Study Quality Assessment Tools for controlled intervention studies and before-and-after (pre-post) studies with no control group were used (31). These tools assess the following relevant bias domains for controlled and uncontrolled studies (30): confounding (e.g., randomization and similarity of groups at baseline), selection bias (i.e., treatment allocation), performance and detection bias (e.g., study blinding and outcome measure assessment), attrition bias (e.g., dropout, adherence, and use of intention-to-treat analysis), and reporting bias.

### Data Synthesis

Data on study characteristics and the critical appraisal of the literature were summarized using a narrative approach and presented in comparative tables. A statistical approach to synthesize results of the included studies (i.e., meta-analysis) was deemed appropriate for studies that reported similar comparisons for outcomes within a given time frame (see eligibility criteria for study outcomes). Data were converted to an appropriate format for inclusion in the meta-analysis; for instance, standard deviations were obtained from standard errors, confidence intervals, or *p*-values (30). The measures of effect size were standardized mean difference—Cohen's *d*—or relative risk, with 95% confidence intervals for continuous or dichotomous outcomes, respectively. The main outcome for this study was short-term reductions in depressive symptoms.

Recent developments in meta-analytical techniques were considered while calculating effect size measures. Doi et al. (32) determined that random effects models may underestimate statistical error, such that a significant proportion of supposed between-study heterogeneity may result from differences in study quality. On this basis, a quality effects estimator for meta-analyses of heterogeneous studies was utilized in this analysis. This estimator uses information collected during the critical appraisal of included studies (favoring larger trials with better quality) and is also robust to subjectivity in quality assessment (32). Whenever data was available, subgroup analyses were made according to timing (e.g., short, mid, and long-term results), and sensitivity analyses were conducted by type of intervention. Publication bias was assessed using the Doi plot and LFK index; specifically, the presence of asymmetry was deemed an indicator of publication bias (33). These analyses were implemented using MetaXL, a free meta-analysis software (34).

## Results

### Study Selection

**Figure 1:**
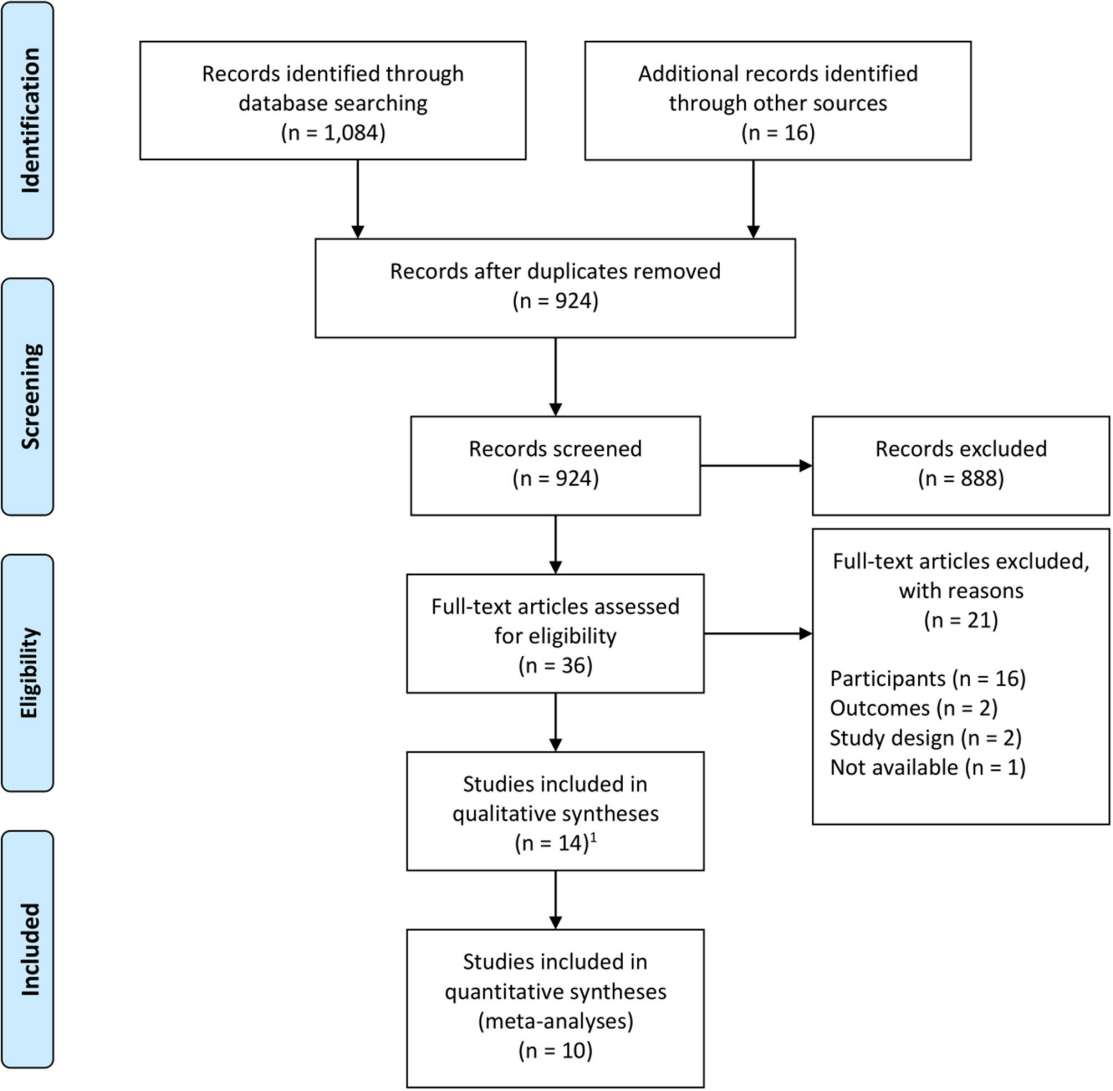
Flow diagram. ^1^The 14 included studies were reported in 15 articles.

Figure 1 presents a flow diagram of the articles identified and reviewed during the different phases of the systematic review and meta-analysis. After initial identification and removal of duplicate records, the titles and abstracts of 924 articles were screened; the full text of the 36 articles deemed eligible in principle were reviewed, leading to the inclusion of 14 studies (published in 15 articles). The meta-analysis for the main outcome—i.e., short-term effect of interventions vs. comparators in reductions of depressive symptoms, in randomized controlled trials—included 6 studies.

### Study Characteristics

Study characteristics are synthesized in Table 1. Of the 14 studies included in this systematic review, 8 were randomized controlled trials (35, 37, 39, 41, 42, 44, 46–48), 3 were controlled before-and-after studies (40, 43, 49), and 3 were uncontrolled before-and-after studies (36, 38, 45). Most of the studies came from developed countries, mainly the United States (35, 36, 38, 41, 42, 44–46), with the exception of one Chilean study, reported in Vitriol et al. (47, 48).

**Table 1:** Characteristics of included studies.

**Study**	**Study design and participants**	**Intervention**	**Comparator**	**Outcomes**
Bradley and Follingstad (35) (USA)	RCT. 49 incarcerated women (34–54 years old, 62% African American), survivors of CSPA, with significant impairment in 2+ TSI subscales or 1 TSI subscale + BDI score ≥ 18; mean baseline BDI score = 22.7	Group therapy (18 sessions, 2.5 hrs each) based on a two-stage model of trauma Tx, including DBT skills (9 sessions) and writing assignments (9 sessions). *N* = 24	No contact (i.e., no Tx). N = 25	A week post-intervention. Depressive symptoms (BDI)
Cameron et al. (36) (USA)	UBA. 92 adults (18–75 years old, 76.1% female) recruited from local communities, with persisting distress due to ACE (85% had 2+ ACE), mean baseline CES-D score = 2.1	ACE Overcomers Program of 12 weekly, 2 hr, sessions providing psychoeducation and skills training, along with a workbook. Participants received a faith-based or secular version of this program. *N* = 92	NA	Post-intervention (at 12 w.). Depressive symptoms (CES-D)
Ha et al. (37) (KOR)	RCT. 33 university students (19–26 years old, 90.6% female), survivors of sexual abuse, mean baseline BDI-II score = 20.8	Four individual writing sessions (30 min) of forgiveness therapy, lasting an average of 2 w, guided by a trained clinical psychologist. *N* = 17	No Tx. *N* = 16	Post-intervention (at 2 w.). Depressive symptoms (BDI-II)
Kimbrough et al. (38) (USA)	UBA. 27 adult survivors of CSA (21 + years old, 89% female) with psychological distress (BSI/GSI ≥ 0.50; mean baseline BDI score = 22.1), in concurrent psychotherapy	MBSR classes plus home practice: 8 weekly, 2.5–3 hr. classes, and a 5 hr. silent retreat; led by a highly experienced MBSR teacher. Plus, three refresher classes over 4 mo. period post-intervention. *N* = 27	NA.	Post-baseline (4, 8, 12, and 24 w.). Depressive symptoms (BDI-II)
Minelli et al. (39) (ITA)	RCT. 26 hospitalized patients (mean age = 52.8 years old; 72.7% female) with TRD (DSM-IV, SCID-I) and 3+ TE (including CT). Mean baseline BDI-II score = 34.5	EMDR (3 individual sessions per w., 60 min., 8 w.), provided by an experienced therapist, plus pharmacotherapy TAU. *N* = 15	TF-CBT (3 individual sessions per w., 60 min., 8 w.), provided by an experienced therapist, plus pharmacotherapy TAU. *N* = 11	Post-randomization (4, 8, 12, and 24 w.). Depressive symptoms and depression response (MADRS, BDI-II)
Morgan and Cummings (40) (CAN)	CBA. Self-referred 80 depressed women (19–63 years old; mean baseline BDI score = 18.5), survivors of CSA	Seven therapy groups (20 w.-program), to reprocess blame and emotional responses to trauma, provided by trained psychotherapists with weekly sup. *N* = 40	No Tx. *N* = 40.	Post-intervention and 3 mo. follow-up. Depressive symptoms (BDI)
Nemeroff et al. (41) (USA)	RCT (*post-hoc* analysis). 315 patients (18–75 years old) with chronic forms of MDD (DSM-IV), HRSD_24_ ≥ 20, and childhood trauma (CTS)	CBASP during 6 w. (twice-weekly sessions, 1–4 w.; weekly sessions, 5–12 w.), focused on remedying maladaptive patterns of interpersonal behavior. *N* = 105 Combination therapy (CBASP + Nefazodone) during 6 w. *N* = 110	Nefazodone during 6 w., initial dose 200 mg/d, increments by 100 mg/d per w. up to a maximum of 600 mg/d. *N* = 100	Post-intervention (at 12 w.). Depression response and remission (HRSD_24_)
Pandina et al. (42) (USA)	RCT (*post-hoc* analysis), with run-in period. 141 depressed adults (18–65 years old; 77.3% female; MDD [DSM-IV], CGI-S ≥ 4, CDS ≥ 20) with insufficient response to AD monotherapy. Mean baseline HRSD_17_ score = 25.1	Risperidone augmentation therapy (up to 1 mg/day) during six w. Patients with insufficient response could increase to 2 mg/day or discontinue double-blind phase. *N* = 73	Matching placebo during six w. *N* = 68	Post-randomization (1, 2, 4, and 6 w.). Depressive symptoms, and depression response and remission (HRSD_17_)
Saxe and Johnson (43) (CAN)	CBA. 69 women (18+ years old) with a history of childhood incest, not living in the house of the perpetrator, in individual therapy referred for group therapy, mean baseline BDI score = 25.3	“Victim to Survivor Group,” a 20-w (2.5 hrs./w) group treatment program, provided by two female co-therapists in a community-based mental health setting. *N* = 38	WL. *N* = 31	Post-intervention (at 20 w.); and follow-up (6 mo.), for the intervention group only. Depressive symptoms (BDI, CES-D)
Scheck et al. (44) (USA)	RCT. 60 traumatized young women (16–25 years old; 90% victims of childhood physical/emotional abuse) with dysfunctional behaviors (e.g., multiple sex partners), mean baseline BDI score = 25.1	Standard EMDR protocol: two 90 min sessions, once a w., provided by trained therapists. *N* = 30	Rogerian-based AL control, provided in two 90 min sessions, once a w., by experienced therapists. *N* = 30	Post-intervention (at 2 w.), and 3 mo. follow-up. Depressive symptoms (BDI)
Talbot et al. (45) (USA)	UBA. 25 depressed women (mean age = 31 years old; MDD [DSM-IV, SCID-I]) with a history of CSA (SCILE), seeking treatment in a community mental health center. Mean baseline BDI score = 29.9	14 weekly session of IPT followed by biweekly sessions + monthly sessions according to clinical indication, provided by trained therapist. *N* = 25	NA	Post-baseline (10, 24, and 36 w.). Depressive symptoms (BDI-II, HRSD_17_)
Talbot et al. (46) (USA)	RCT. 70 depressed women (mean age = 36 years old; MDD [DSM-IV, SCID-I]) with a history of CSA (SCILE), seeking treatment in a community mental health center. Mean baseline BDI-II score = 34.6	A 16-session IPT program conducted over a period of 36 weeks, provided by trained therapists (weekly sup.). *N* = 37	UC psychotherapy (excluding IPT), provided by therapists with weekly sup. *N* = 33	Post-randomization (10, 24, and 36 w.). Depressive symptoms (BDI-II, HRSD_17_)
Vitriol et al. (47), and Vitriol et al. (48) (CHL)	RCT. 87 depressed women (20+ years old; ICD-10 and HRSD_21_ ≥ 21) with a history of CT (BPSAQ > 2), referred to a regional hospital from PHC clinics. Mean baseline HRSD_21_ score = 34.3	Brief structured TF psychodynamic psychotherapy (3 mo.), delivered by a psychologist (weekly sup.). Pharmacotherapy as usual. Monthly appointment with psychiatrist and follow-up with social worker. *N* = 44	UC for depression (including pharmacotherapy), according to clinical guidelines. Psychotherapy (not TF), upon request. *N* = 43	Post-randomization (3 and 6 mo.). Depressive symptoms, and depression response and remission (HRSD_21_)
Westbury and Tutty (49) (CAN)	CBA. 32 moderately to severely depressed women in their mid-thirties (mean baseline BDI score = 23.8), with at least 6 mo. of psychotherapy for CSA	Brief structured group psychotherapy (2 hrs, 10–12 w, 6–8 participants), with body-focused relaxation and personal boundary exercises. *N* = 22	WL. *N* = 10	Post-intervention (at 10–12 w). Depressive symptoms (BDI)

*Age at which sexual abuse occurred not specified in Ha et al. (37). Data for mean baseline depression scores in Bradley and Follingstad (35), Cameron et al. (36), Minelli et al. (39), Scheck et al. (44) reported for treatment completers only. Data for mean baseline depression scores in Saxe and Johnson (43) reported for the analyzed cases only. In Nemeroff et al. (41), reported sample size corresponds to treatment completers who had chronic forms of depression and childhood trauma; the study compared three interventions, and antidepressant treatment alone was chosen as the comparator; and, the estimated sample size of completers who had chronic forms of depression and childhood trauma in each intervention were calculated assuming equal drop-out rates between study arms. Sample characteristics in Ha et al. (37), Minelli et al. (39), Morgan and Cummings (40), Saxe and Johnson (43) were only reported for treatment completers*.

*ACE, adverse childhood experiences; AL, active listening; BPSAQ, Brief Physical and Sexual Abuse Questionnaire; CBA, controlled before-and-after study; CBT, cognitive behavioral therapy; CDS, Carroll Depression Scale; CGI-S, Clinical Global Impressions-Severity; CSA, child sexual abuse; CSPA, childhood sexual and/or physical abuse; CT, childhood trauma; CTS, Childhood Trauma Scale; D., day; DSM-IV, diagnostic and statistical manual of mental disorders, fourth edition; EMDR, eye movement desensitization and reprocessing; Hr(s)., hour(s); ICD, International Classification of Diseases; IPT, Interpersonal Psychotherapy; MBSR, mindfulness meditation-based stress reduction; MDD, major depressive disorder; Mins., minutes; Mo., months; NA, not applicable; PHC, primary health care; RCT, randomized controlled trial; SCID-I, Structured Clinical Interview for DSM-IV for Axis I Disorders; SCILE, Structured Clinical Interview of Life Events; Sup., supervision; TAU, treatment as usual; TE, traumatic events; TF, trauma-focused; TRD, treatment-resistant depression; TSI, Trauma Symptom Inventory; Tx, treatment; UBA, uncontrolled before-and-after study; UC, usual care; W., week(s); WL, waiting list*.

*Country codes. CAN, Canada. CHL, Chile. ITA, Italy. KOR, South Korea. USA, United States of America*.

*Outcomes (depression): HRSD, Hamilton Rating Scale for Depression, with subscript indicating number of items of the instrument. BDI, Beck Depression Inventory. BDI-II, BDI second edition. MADRS, Montgomery-Asberg Depression Rating Scale. PaRTS-D, Patient-Reported Troubling Symptoms of Depression. CES-D, Centre for Epidemiological Studies Depression Scale*.

Our systematic review included the results of 1,106 individuals. Total sample sizes for included studies ranged from 25 (45) to 315 (41), made up mostly of female participants of reproductive age, recruited from outpatient settings (41–43, 45, 46, 49). The majority of the studies used self-report questionnaires to ascertain early adverse stress, and the most frequent was the Childhood Trauma Questionnaire (45, 46). Seven interventions were specifically aimed at victims of childhood sexual abuse (37, 38, 40, 43, 45, 46, 49). Depressive status of the participants was defined as an eligibility criterion in eight of the fourteen studies (35, 38, 39, 41, 42, 45–48), and six of these studies used diagnostic clinical interviews to determine the presence of major depressive disorder (39, 41, 42, 45–48). No studies reported the inclusion of patients with bipolar disorder. All of the studies employed self-reported questionnaires for the assessment of depressive symptoms, with baseline values in the ranges of moderately severe to severe depressive symptoms.

Interventions were categorized as psychological (35–38, 40, 43–46, 49), pharmacological (42), or combined treatment (i.e., psychological and pharmacological interventions) (39, 41, 47, 48). Regarding the psychological interventions, three studies compared psychological interventions against no intervention (35, 37, 40), two studies compared psychological interventions against waiting-lists (43, 49), and two studies compared psychological interventions against control psychotherapies (44, 46). Of the three uncontrolled studies, all assessed psychological interventions (36, 38, 45). Further details of the content of the psychological interventions are shown in Table 2; most of these conceptualized current interpersonal difficulties as a consequence of early adverse stress, provided a supportive and safe environment to talk about traumatic experiences, and enhanced personal skills and resources. Pandina et al.'s randomized controlled trial was the only study classified as a pharmacological intervention and tested the efficacy of risperidone augmentation therapy vs. matching placebo (42). Finally, three studies reported combined treatments: Minelli et al.'s randomized controlled trial compared the effectiveness of two active psychotherapies plus psychopharmacological treatment as usual (39); Nemeroff et al.'s (41) was a three-arm randomized controlled study testing the efficacy of nefazodone, an integrative model of psychotherapy, and combination therapy; and Vitriol et al.'s study compared a trauma-focused psychotherapy plus pharmacological treatment as usual with usual care for the management of depression (including pharmacological treatment) (47, 48).

**Table 2:** Synthesis of content of psychological interventions included.

**Study**	**Contents/focus of psychological interventions**
Bradley and Follingstad (35)	*Group therapy*. Patients received education about interpersonal victimization and affect regulation and were encouraged to write meaningful narratives of their life experiences and to make connections between past life events and current feelings. Group sessions were focused on salient issues for incarcerated women and were used to complete and share written experiences
Cameron et al. (36)	*ACE Overcomers Program*. Patients received education on ACEs and their biopsychosocial sequelae. Non-judgmental self-examination and learning skills for emotion regulation and resilience were encouraged. Patients were given a workbook summarizing weekly lessons and homework assignments. The faith-based version used biblical examples, and the secular version employed quotations from well-known scholars
Ha et al. (37)	*Forgiveness writing therapy*. Guided by a trained clinical psychologist, patients were encouraged to wrote detailed accounts of their sexual abuse and their thoughts and feelings about the event, to emphasize self-acceptance by consoling the wounded self, reassessing the traumatic event, and reconstructing life values
Kimbrough et al. (38)	*Mindfulness meditation-based stress reduction program*. Meditation practices were introduced in four formats: sitting meditation, progressive body awareness, contemplative walking, and gentle yoga stretching. Formal and informal daily mindfulness practices and completion of a workbook were encouraged. Participants were reminded to stay present and trained in positive growth awareness
Minelli et al. (39)	*Trauma-focused cognitive behavioral therapy* provides psychoeducation, anxiety management skills, *in vivo* and/or imaginal exposure, and cognitive restructuring. *Eye movement desensitization and reprocessing* protocol, where individuals focus on a traumatic image, thought, emotion and bodily sensations whilst receiving bilateral stimulation (e.g., eye movements) to reprocess a traumatic memory
Morgan and Cummings (40)	*Group therapy*. Patients were encouraged to examine their abuse experiences in a safe environment using a societal framework, under which they were allowed to place the blame on their abusers and to express their anger in healthy ways. Education was provided on common responses to trauma and coping mechanisms
Nemeroff et al. (41)	*Cognitive behavioral analysis system of psychotherapy*. Integrative therapy helping patients to generate empathic behavior, identify and change interpersonal patterns related to depression, and heal interpersonal trauma, which combines problem solving techniques, examination of past traumatic experiences and differentiation of those from healthier relationships, and behavioral skills training
Saxe and Johnson (43)	*Victim to Survivor Group*. Intervention that seeks to establish a safe, supportive, and nonthreatening place to explore and recount each member's own history of incest, and its past and current effects, and focuses on progress made by each group member toward achieving her personal goals.
Scheck et al. (44)	A standard *eye movement desensitization and reprocessing* protocol as previously described in Minelli et al. (39)
Talbot et al. (45) and Talbot et al. (46)	Manualized *interpersonal psychotherapy* focused on one of four interpersonal problem areas (grief, interpersonal disputes, role transitions, and interpersonal patterns of deficit), helping patients to better communicate their interpersonal needs. Although the focus was on current interpersonal difficulties, the influence of traumatic experiences upon these conflicts was explored
Vitriol et al. (47) and Vitriol et al. (48)	*Brief psychodynamic psychotherapy* focused on developing cognitive understanding of personal characteristics and behaviors that allowed the repetition of traumatic experiences in the present. Sexual trauma was validated, and behavioral and emotional responses (i.e., guilt and shame) were addressed to prevent new situations of abuse
Westbury and Tutty (49)	*Women's survivor groups*, based on a body-focused feminist model, that provide a supportive and safe environment to address childhood sexual abuse, with activities including body-focused relaxation techniques, personal boundaries exercises, problem-solving strategies, development of interpersonal and life management skills, and setting personal and societal goals

*ACE, adverse childhood experiences*.

All the included studies, except for Nemeroff et al. (41), provided outcome data on depressive symptoms. Four studies assessed response to treatment of depression (39, 41, 42, 47, 48), and three reported information on remission of depressive symptoms (39, 41, 42, 47, 48). Short-term evaluations of depression outcomes were the most commonly reported results; only Bradley and Follingstad (35), Morgan and Cummings (40), and Saxe and Johnson (43) did not include a short-term assessment. Four self-report questionnaires were used to assess depressive symptoms (ordered by frequency): the Beck Depression Inventory (*n* = 10), the Hamilton Rating Scale for Depression (*n* = 5), the Center for Epidemiological Studies Depression Scale (*n* = 2), and the Montgomery-Asberg Depression Rating Scale (*n* = 1).

### Risk of Bias Within Studies

Syntheses of the critical appraisal of each of the studies are presented in Tables 3, 4, for controlled intervention studies and for before-after studies with no control group, respectively. No single study met all the quality criteria. Although Bradley and Follingstad (35), Ha et al. (37), Talbot et al. (46), and Vitriol et al. (47, 48) reported using random assignment, the use of appropriate random-sequence generation and allocation concealment strategies could not be determined. Blinding of patients and providers was not possible for studies testing psychological interventions. In addition, the use of blinded outcomes assessors could not be determined, based on the information provided in Bradley and Follingstad (35), Ha et al. (37), Saxe and Johnson (43), and Westbury and Tutty (49). In the same vein, Bradley and Follingstad (35), Ha et al. (37), and Nemeroff et al. (41) did not report enough information to ascertain whether groups of individuals with early adverse stress were equivalent on baseline characteristics. In the *post-hoc* analyses by Nemeroff et al. (41) and Pandina et al. (42), dropout rates and adherence to treatment protocols were not reported. Most studies did not include or state power calculations, which may have increased the risk of underpowered results, with the exception of Cameron et al. (36), Kimbrough et al. (38), and Talbot et al. (46). Additionally, 4 of the 11 controlled intervention studies analyzed data on an intention-to-treat basis (42, 46–49), while the rest used per protocol or completers analyses. Studies without a control group did not use an interrupted time series design (36, 38, 45), which may have increased confidence in the accurate assessment of study outcomes.

**Table 3:** Critical appraisal for controlled intervention studies.

**Study**	**# Questions of the Study Quality Assessment Tool for controlled intervention studies**													
	**1**	**2**	**3**	**4**	**5**	**6**	**7**	**8**	**9**	**10**	**11**	**12**	**13**	**14**
**RCTs**														
Bradley and Follingstad (35)	+	CD	CD	–	CD	CD	–	–	+	+	+	–	+	–
Ha et al., (37)	+	CD	CD	–	CD	CD	+	+	+	+	+	–	+	–
Minelli et al. (39)	+	+	+	–	+	+	+	–	+	+	+	–	+	–
Nemeroff et al. (41)	+	+	+	–	+	CD	CD	CD	CD	+	+	–	–	–
Pandina et al. (42)	+	+	+	+	+	+	CD	CD	CD	+	+	–	–	+
Scheck et al. (44)	+	+	+	–	–	+	–	+	+	+	+	–	+	–
Talbot et al. (46)	+	CD	CD	–	–	+	–	–	–	+	+	+	+	+
Vitriol et al. (47) and Vitriol et al. (48)	+	CD	CD	–	+	+	+	–	+	+	+	–	+	+
**CBAs**														
Morgan and Cummings (40)	–	NA	NA	–	–	–	–	–	+	CD	+	–	+	–
Saxe and Johnson (43)	–	NA	NA	–	CD	+	+	+	+	+	+	–	+	–
Westbury and Tutty (49)	–	NA	NA	–	CD	–	+	+	CD	+	+	–	+	+

*Topics covered by the questions for the Study Quality Assessment Tool for controlled intervention studies: (1) randomized study; (2) adequate randomization; (3) allocation concealment; (4) blinding of study participants; (5) blinding of outcome assessors; (6) similarity of groups at baseline; (7) overall dropout rate; (8) differential dropout rate; (9) adherence to treatment protocols; (10) avoidance of other interventions; (11) outcome measures assessment; (12) power calculation; (13) prespecified outcomes; and (14) intention-to-treat analysis. (+): quality criteria accomplished; (–): quality criteria not accomplished; CD: could not be determined; NA: not applicable. In Nemeroff et al. (41), adequate randomization, allocation concealment, and blinding were critically appraised according to the details provided in the original study (50). As Nemeroff et al. (41) and Pandina et al. (42) reported post-hoc analyses, the prespecified outcomes criterion was classified as not accomplished*.

**Table 4:** Critical appraisal for before-after studies with no control group.

**Study**	**# Questions of the Study Quality Assessment Tool for before-after (pre-post) studies with no control group**											
	**1**	**2**	**3**	**4**	**5**	**6**	**7**	**8**	**9**	**10**	**11**	**12**
Cameron et al. (36)	+	+	+	–	+	+	+	+	–	+	–	NA
Kimbrough et al. (38)	+	+	+	+	+	+	+	CD	+	+	–	NA
Talbot et al. (45)	+	+	+	+	–	–	+	–	–	+	–	NA

*Topics covered by the questions for the Study Quality Assessment Tool for before-after (pre-post) studies with no control group: (1) study question; (2) eligibility criteria and study population; (3) sample representativeness; (4) all eligible participants are enrolled; (5) power calculation; (6) intervention clearly described; (7) outcome measures assessment; (8) blinding of outcome assessors; (9) follow-up rate; (10) statistical analysis; (11) multiple outcome measures; (12) group-level interventions and individual-level outcome effort. (+): quality criteria accomplished; (–): quality criteria not accomplished; CD: could not be determined; NA: not applicable*.

### Syntheses of Results

Syntheses of results are outlined below and presented in forest plots or tables for depressive symptoms, response to treatment of depression, and remission of depressive symptoms. Whenever available, results were further classified according to timing (short, mid, and long-term results). This presentation is complemented with details on intervention comparisons made in the included studies (e.g., psychological interventions against control psychotherapies, or psychological interventions vs. no intervention). In the case of uncontrolled before-and-after studies, pre-post effects of interventions were synthesized.

### Depressive Symptoms

**Figure 2:**
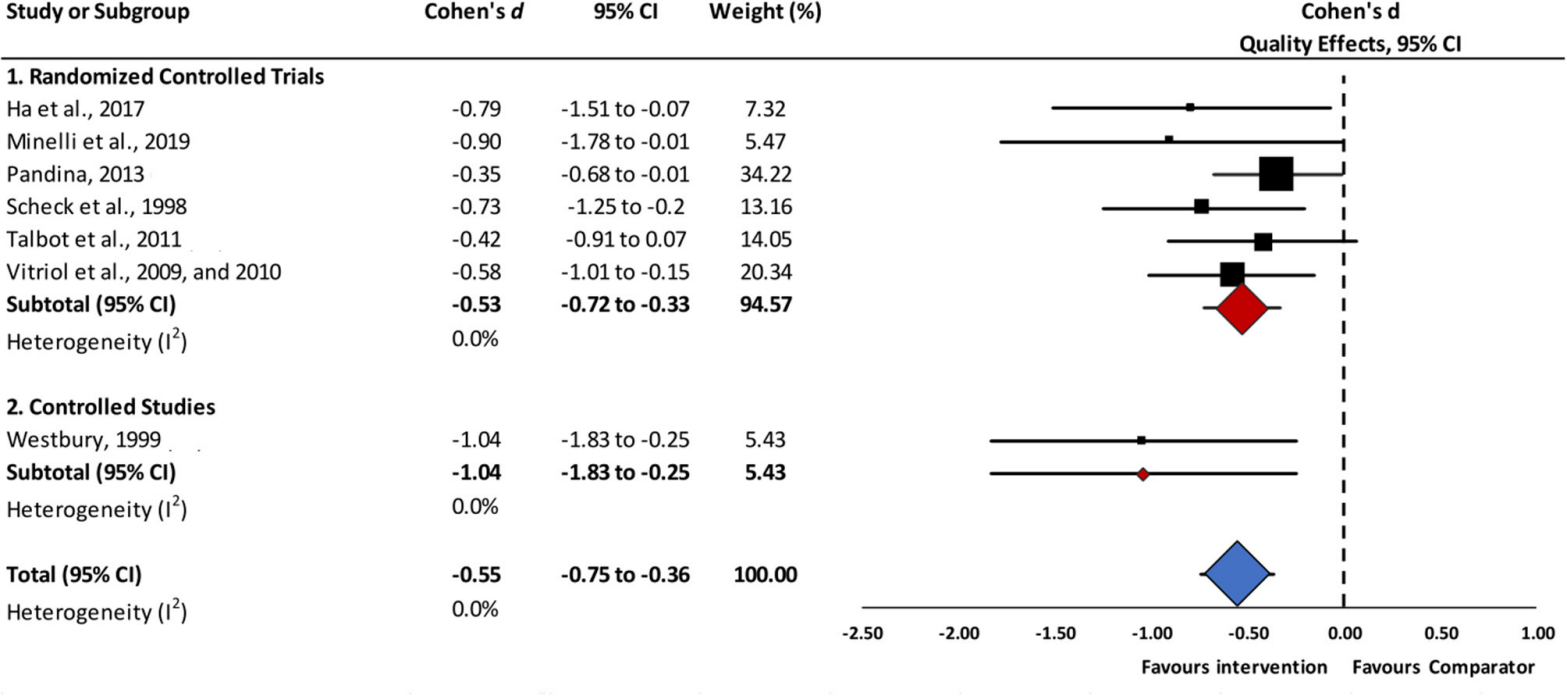
Short-term effects of interventions vs. comparators in reductions of depressive symptoms. *Abbreviations*. 95% CI, 95% confidence interval. Short-term effects: assessment up to 12-weeks follow-up.

Meta-analysis for short-term (i.e., up to 12-week follow-up) reductions in depressive symptoms showed a statistically significant moderate effect size favoring intervention groups vs. comparators (*d* = −0.55, 95% CI −0.75 to −0.36, seven studies, *I*^2^ = 0%) (Figure 2). Two randomized controlled trials testing psychological interventions against control psychotherapies (44, 46) yielded favorable results for the intervention groups (*d* = −0.57, 95% CI −0.93 to −0.21, *I*^2^ = 0%). Complementarily, with respect to the combined interventions, a moderate to large effect size favored eye movement desensitization plus pharmacological treatment as usual vs. trauma-focused cognitive behavioral psychotherapy plus pharmacological treatment as usual, although with a wide confidence interval (*d* = −0.90, 95% CI −1.78 to −0.01) (39), and Vitriol et al.'s trauma-focused intervention plus usual pharmacotherapy vs. usual care for depression yielded a moderate effect size in favor of the intervention group (*d* = −0.58, 95% CI −1.01 to −0.15) (47, 48). In terms of pharmacological treatments, in Pandina et al.'s study (42), risperidone augmentation therapy compared to matching placebo was efficacious in the short-term improvement of depressive symptoms, with a small effect size and wide confidence interval (*d* = −0.35, 95% CI −0.68 to −0.01). Finally, a non-statistically significant moderate-to-large effect size was found for pre-post reductions on depressive symptoms in the three uncontrolled before-and-after studies (*d* = −0.76, 95% CI −1.67 to 0.14, *I*^2^ = 86.4%) (36, 38, 45). Further exploration of heterogeneity revealed that Cameron et al. (36) was the only study reporting non-statistically significant pre-post differences (*d* = −0.33, 95% CI −0.70 to 0.03).

**Figure 3:**
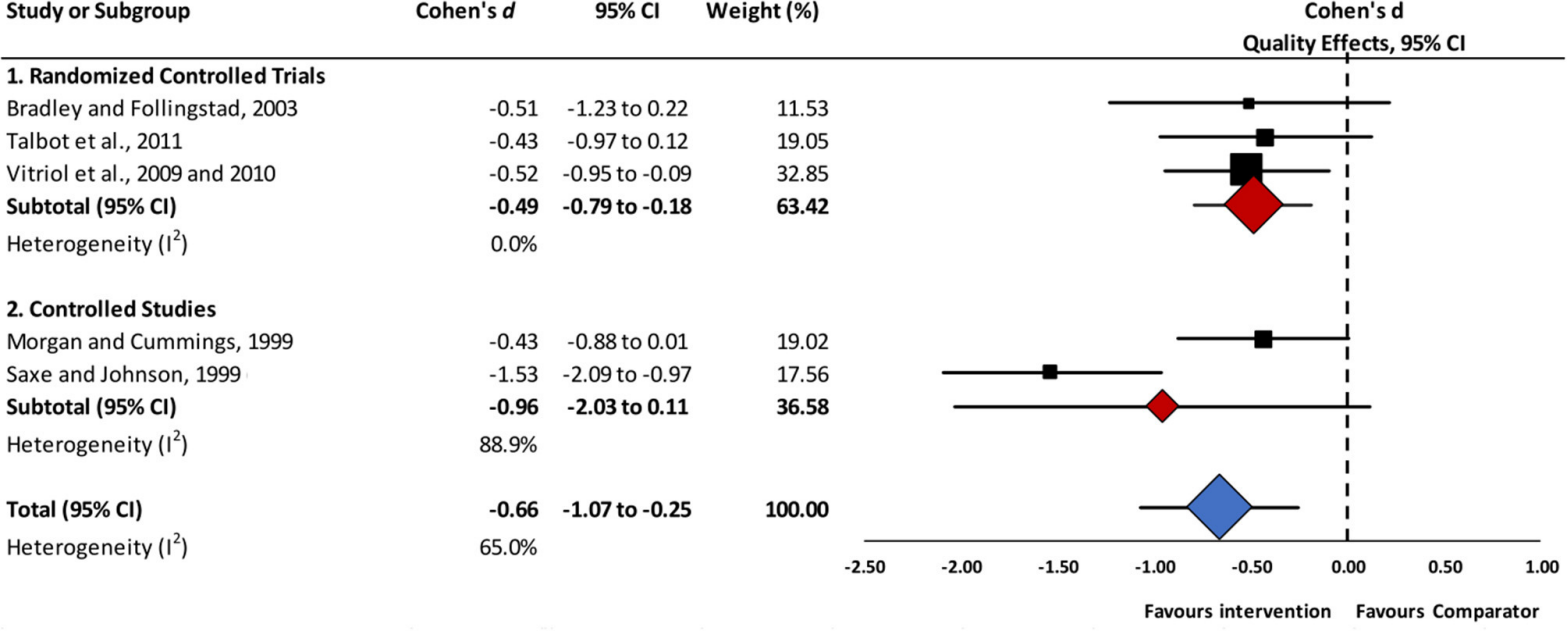
Mid-term effects of interventions vs. comparators in reductions of depressive symptoms. *Abbreviations*. 95% CI, 95% confidence interval. Mid-term effects: assessment up to 24-weeks follow-up.

Results of a meta-analysis for mid-term (i.e., assessments carried out between 12- and 24-weeks follow-up) reductions in depressive symptoms, are displayed in Figure 3. In this analysis, which included five studies, intervention groups achieved statistically significant lower depressive symptoms scores, with moderate effect sizes, compared to controls (*d* = −0.66, 95% CI −1.07 to −0.25, *I*^2^ = 65.0%). Randomized controlled trials, whether comparing a psychological intervention to no intervention (35), a psychological intervention with a control psychotherapy (46), or a combined intervention vs. usual care (47, 48), yielded consistent, non-heterogeneous estimates in favor of these interventions (*d* = −0.49, 95% CI −0.79 to −0.18, *I*^2^ = 0%). In contrast, the statistical synthesis of psychological interventions in two controlled before-and-after studies was highly heterogeneous (*I*^2^ = 88.9%); thus, study results must be interpreted individually (40, 43). Finally, regarding the synthesis of uncontrolled before-and-after studies (38, 45), a statistically significant pre-post reduction in depressive symptoms, with a large effect size, was observed (*d* = −0.82, 95% CI −1.25 to −0.39, *I*^2^ = 0%).

Long-term (assessment after at least 25-week follow-up) reductions in depressive symptoms were assessed in two studies that evaluated psychological interventions, with a randomized controlled trial reporting no differences between groups (*d* = −0.34, 95% CI −0.89 to 0.21) (46), and an uncontrolled before-and-after study finding statistically significant pre-post reductions in depressive symptoms, with a large effect size (*d* = −1.04, 95% CI −1.75 to −0.33) (45).

Sensitivity analyses found that psychological and combined treatments reported higher effect sizes for reductions in depressive symptoms (*d* = −0.56, 95% CI −0.82 to −0.30; and *d* = −0.59, 95% CI −0.87 to −0.30, respectively) than the single study assessing a pharmacological intervention (*d* = −0.35, 95% CI −0.68 to −0.01), although the overlapping confidence intervals for these estimations revealed that there were no statistically significant differences between the subgroups.

### Response to Treatment of Depression

A meta-analysis for short-term response to depression treatment was not feasible, given the markedly different nature of comparisons across the three studies (39, 42, 47, 48). Statistically significant differences were only found for Pandina et al.'s pharmacological intervention study (42), which demonstrated a higher likelihood of short-term response to depression treatment favoring the intervention group (Table 5). Vitriol et al.'s trial (47, 48), which tested a combined intervention, was the only study to report data for mid-term response to treatment, thought they did not find statistically significant differences between study groups (*relative risk* = 1.50, 95% CI 0.83 to 2.73).

**Table 5:** Short-term response to treatment and remission of depressive symptoms.

**Study**	**Intervention**		**Comparator**		**Relative Risk**	**95% CI**
	**Cases**	***N***	**Cases**	***N***		
**Response**						
Minelli et al. (39)	10	12	5	10	1.67	0.85–3.26
Pandina et al. (42)	29	71	15	65	1.77	1.05–2.99
Vitriol et al. (47) and Vitriol et al. (48)	13	39	6	40	2.22	0.94–5.26
**Remission**						
Nemeroff et al. (41)	110	215	33	100	1.55	1.14–2.11
Pandina et al. (42)	14	71	6	65	2.14	0.87–5.23
Vitriol et al. (47) and Vitriol et al. (48)	2	39	1	40	2.05	0.19–21.72

*95% CI, 95% confidence interval*.

*Nemeroff et al. (41) compared three interventions, and antidepressant treatment alone was chosen as the comparator; sample size was estimated for completers who had chronic forms of depression and childhood trauma in each intervention group, assuming equal drop-out rates between study arms*.

### Remission of Depressive Symptoms

Short-term remission of depressive symptoms was assessed in three studies (41, 42, 47, 48); of theses, Nemeroff et al.'s combined intervention study (41) was the only to demonstrate a higher likelihood of remission of depressive symptoms, favoring the intervention group against nefazodone alone (*relative risk* = 1.55, 95% CI 1.14 to 2.11). The only study providing information on mid-term remission of depressive symptoms, which was a combined intervention (47, 48), found no statistically significant differences between study groups (*relative risk* = 3.89, 95% CI 0.89 to 17.05).

### Risk of Bias Across Studies

**Figure 4:**
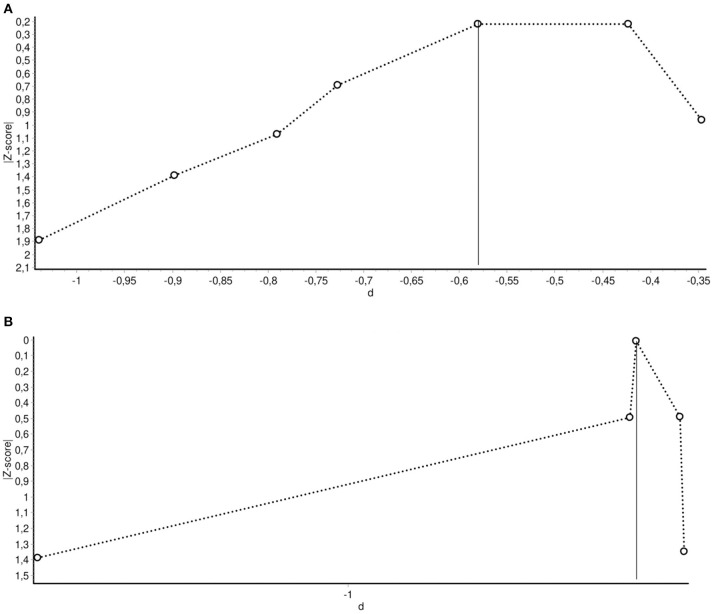
**(A)** Doi plot for the assessment of publication bias in studies assessing short-term effects of interventions in reducing depressive symptoms. LFK index: −2.56 (major asymmetry). **(B)** Doi plot for the assessment of publication bias in studies assessing mid-term effects of interventions in reducing depressive symptoms. LFK index: −1.10 (minor asymmetry).

The Doi plots for publication bias showed major (LFK index = −2.56) and minor (LFK = −1.10) asymmetry in meta-analyses for short- and mid-term reductions of depressive symptoms, respectively (Figures 4A,B). These findings might provide unequivocal evidence for publication bias, implying that studies with negative (i.e., favoring controls) or equal outcomes are lacking. However, these findings might also be attributable to chance, given the few number of studies included in the analyses.

## Discussion

### Summary of Main Findings

Our systematic review included eight randomized controlled trials, three controlled before-and-after studies, and three uncontrolled before-and-after studies, representing 1,106 participants with depression. No study included patients with bipolar disorder. The syntheses of results suggest that psychological, pharmacological, and combined treatment interventions in adults with depression and a history of early adverse stress may be effective in reducing depressive symptoms in the short- and mid-term, with moderate effect sizes. Sensitivity analyses found that psychological or combined treatment interventions had greater effect sizes than pharmacological interventions, although with no statistically significant differences. In controlled or uncontrolled before-and-after studies of psychological interventions, a trend favoring a significant reduction of depressive symptoms was also observed; however, these estimates had substantial heterogeneity and, in some cases, did not achieve statistical significance. Assessments of long-term outcomes were only reported for a small number of studies, as was also the case for symptoms response and remission of depression in the short- and mid-term; these assessments had mixed results that, in general, could not be statistically synthesized.

### Overall Completeness and Applicability of Evidence

The evidence synthesized in this systematic review may only be relevant for depressed adults with a history of early adverse stress. Moreover, as most of studies were conducted in developed countries, with samples with unclear or limited ethnic diversity, the results of this review may not be applicable to other locations, such as low- or middle-income countries, or in minority populations. Half of the studies were aimed at victims (mostly women) of childhood sexual abuse (37, 38, 40, 43, 45, 46, 49), with an important component of the psychological interventions addressing this early adverse stressor. On the other hand, most of the studies in our review reported psychological interventions; there is limited available evidence on pharmacological or combined treatment interventions for depressed individuals with a history of exposure to early adverse stress. There is also scarce evidence on long-term treatment outcomes, even for psychological interventions. Finally, although the body of reviewed literature dates to more than a decade ago, on average, the fact that three studies were recently published (36, 37, 39) may be a sign of a renewed interest in this research topic.

### Quality of the Evidence

The summary of findings of this systematic review and meta-analysis must be interpreted with considerable caution due to the limitations observed in the design and implementation of the included studies. The included studies with higher methodological quality (i.e., randomized clinical trials) also suffered from potentially relevant sources of bias, such as selection bias due to unclear randomization procedures and, in some cases, lack of information to ensure equivalence between the baseline characteristics of study groups. Likewise, the risk of performance and detection bias could have been present in studies evaluating psychological interventions that relied on the use of patient self-reported outcomes, without clearly stating the use of blinded outcome assessment. Only a minority of studies used intention-to-treat analysis, a useful strategy to limit attrition bias. Finally, the asymmetry detected in the Doi plots were consistent with a high probability of publication bias, in favor of studies with positive results.

### Potential Biases in the Review Process

In addition to the study and outcome-level limitations outlined above, attention should be paid to the strengths and weaknesses of the review process. The prospective registration of the protocol, the usage of the PRISMA statement reporting guidelines (29), and the implementation of this systematic review and meta-analysis according to the standards set by the Cochrane Collaboration (30) were important elements to ensure transparency and quality throughout the review process. The exploration of the published studies in multiple databases and in the reference lists of the included studies guaranteed a reasonable extension of the search process. The data extraction and study quality assessments were carried out independently by two researchers, with several and periodical quality checks. Notwithstanding, this review did not consider gray literature and a relevant source of information in mental health research (PsycINFO). Moreover, subsequent assessments of the asymmetry in the meta-analyses were consistent with the existence of publication bias, suggesting there is a high likelihood that negative results from other studies have simply not been published. Additionally, although the exclusion of studies published in languages other than English or Spanish may have biased the reviewing process, very few records were excluded for this reason. Furthermore, some of the estimates had to be extracted from unadjusted or poorly reported data, which may have affected the precision and bias of the estimated effect of the interventions (30). Importantly, the small sample of studies found coupled with their relative clinical diversity, are major limitations to conclusively judge the effectiveness of the reviewed interventions.

### Comparison With Previous Studies

This is the first systematic review and meta-analysis to provide direct evidence on the effectiveness of treatment interventions for depressive or bipolar disorders in adults with a history of early adverse stress. A previous synthesizing effort, by Nanni et al. (18), compared the response to treatment in depressed patients with or without childhood maltreatment. The aforementioned study found that maltreated and depressed individuals were at higher risk for poor response to treatment (18). Nanni et al. also highlighted the need to test new treatments targeting the biological vulnerabilities evidenced in victims of childhood trauma (18, 19). Despite this, the present review did not find studies in this direction, and novel therapeutic strategies that consider the neurobiological mechanisms involved in the differential risk of depression among individuals with early adverse stress, as discussed by Nemeroff, are still required (10).

Beyond the important methodological limitations of the evidence reviewed, the synthesis of studies focused on patients with early adverse stress provides provocative insights. First, the effect sizes for acute symptoms of depression were comparable to those reported by a network meta-analysis that evaluated the effectiveness of different modalities of cognitive behavioral psychotherapy for depression (51), one of the current standards in the management of this mental disorder. Thus, treatment interventions for depression in adults exposed to early adverse stress seems to be an auspicious field for future clinical research. Second, psychological interventions for depression—or the psychological components of combined treatment interventions –that recognize the impact of childhood trauma on interpersonal conflicts and coping strategies in adulthood show promise in achieving better clinical outcomes for this patient population. This is consistent with so-called “trauma-informed care,” which recognizes the ubiquity of early adverse stress, and its burdensome, lingering consequences, and implements strategies to provide a supportive environment to avoid retraumatization (52, 53).

### Implications for Future Research and Clinical Practice

Despite the well-documented long-lasting, negative, and costly impact of early adverse stressors on adult psychopathology, evidence on treatment alternatives remains scant. This is the first systematic review and meta-analysis to provide direct evidence on the effectiveness of interventions for depressed adults with a history of early adverse stress. The results of our systematic review and meta-analysis tentatively suggest that trauma-focused treatment interventions—whether psychological interventions alone or in combination with pharmacotherapy—have the potential to reduce the severity of depressive symptom in adults who were exposed to early adverse stress. We hypothesize that this shared psychological component might partly confer a greater potential for success to interventions aimed at this population, supporting earlier findings by Nemeroff et al. (41), Vitriol et al. (47), and Vitriol et al. (48). Moreover, although an important clinical diversity was found—which might be a major limitation of our review—the consistency of the effects is striking, strengthening the case for the potential usefulness of this type of interventions. Nevertheless, additional intervention studies, specifically targeting depressive or bipolar disorders in adults with early adverse stress, are needed. Of particular concern is the absence of studies including patients with bipolar disorders. In this regard, to date only one published study protocol has reported a clinical trial for patients with bipolar disorder and a history of trauma (54).

On the other hand, we noted a relative lack of studies testing specific pharmacological interventions, to address, for instance, the neurobiological mechanisms outlined by Nemeroff (10). In the same line, although Minelli et al.'s results are suggestive (39), our review found different psychological approaches to early adverse stress in depressed adults which were not possible to compare against each other. More research should also be conducted to study the effectiveness of combined interventions. Additionally, greater efforts should be made to develop and test personalized approaches to treatment co-occurring early adverse stressors, without neglecting interventions addressing specific types of childhood trauma. Furthermore, future investigative efforts should focus on patients' samples in developing countries or in populations of lower social position, where childhood trauma may be more common (55). Finally, forthcoming research should pay particular attention to common and critical sources of bias observed in the studies included in this systematic review, and a strong commitment should be made to publish all trials, even those with negative results.

## Data Availability Statement

The original contributions presented in the study are included in the article/Supplementary Material, further inquiries can be directed to the corresponding author.

## Author Contributions

PM, SG, and PV: study conception and design. PM, DD, AG, and MC: data collection. PM, SG, DD, AG, MC, SS, and PV: analysis and interpretation of data. PM, DD, AG, MC, and SS: drafting of manuscript. SG and PV: critical revision. All authors contributed to the article and approved the submitted version.

## Conflict of Interest

The authors declare that the research was conducted in the absence of any commercial or financial relationships that could be construed as a potential conflict of interest.
