# Supplementary material for: Early Adverse Stress and Depressive and Bipolar Disorders: A Systematic Review and Meta-Analysis of Treatment Interventions
Source: Front Psychiatry. 2021 Apr 26;12:650706. doi: 10.3389/fpsyt.2021.650706 (PMC8107272; doi:10.3389/fpsyt.2021.650706)
Supplement: Supplementary file 1 [file Data_Sheet_1.PDF]

1.1. Search strategies

CINAHL

((MH “affective disorders+”) OR (MH “bipolar disorder+”)) AND ((TI childhood adversities) OR (AB childhood adversities) OR (TI abuse and neglect) OR (AB abuse and neglect) OR (TI early life stress) OR (AB early life stress) OR (TI child maltreatment) OR (AB child maltreatment) OR (TI childhood trauma) OR (AB childhood trauma) OR (TI early life trauma) OR (AB early life trauma) OR (MH “adverse childhood experiences+”) OR (MH “child abuse survivors+”) OR (MH “child abuse+”) OR (MH “life change events+”) OR (MH “psychological trauma+”)) AND ((MH “clinical trials+”) OR (MH “interrupted time series analysis”) OR (MH “controlled before-after studies”) OR (MH “quasi-experimental studies+”))

EMBASE

(‘depression’/exp) AND (‘childhood adversities’:ab,ti OR ‘childhood adversity’/exp OR ‘childhood trauma survivor’/exp OR ‘childhood sexual abuse survivor’/exp OR ‘child abuse’/exp OR ‘psychotrauma’/exp OR ‘abuse and neglect’:ab,ti OR ‘early life stress’:ab,ti OR ‘child maltreatment’:ab,ti OR ‘childhood trauma’:ab,ti OR ‘early life trauma’:ab,ti) AND (‘clinical trial’/exp OR ‘clinical trial (topic)’/exp OR ‘interrupted time series’:ab,ti OR ‘controlled before-after stud*’:ab,ti OR ‘quasi experimental study’/exp)

Pubmed

(((((Depression[Mesh] OR “Depressive Disorder”[Mesh] OR “Bipolar and Related Disorders”[Mesh])))) AND (((“childhood adversities”[Title/Abstract] OR “adverse childhood experiences”[Mesh] OR “adult survivors of child abuse”[Mesh] OR “adult survivors of child adverse events”[Mesh] OR “child abuse”[Mesh] OR “abuse and neglect”[Title/Abstract] OR “early life stress”[Title/Abstract] OR “child maltreatment”[Title/Abstract] OR “childhood trauma”[Title/Abstract] OR “early life trauma”[Title/Abstract] OR “life change events”[Mesh] OR “psychological trauma”[Mesh])))) AND ((“Clinical Trial”[Publication Type] OR “Clinical Trials as Topic”[Mesh] OR “Interrupted Time Series Analysis”[Mesh] OR “Controlled Before-After Studies”[Mesh] OR Quasi-experiment*[Title/Abstract]))

Web of Science

TS=(depression OR “depressive disorder” OR bipolar OR “bipolar and related disorders”) AND TS=(“childhood adversities” OR “adverse childhood experiences” OR “adult survivors of child abuse” OR “adult survivors of child adverse events” OR “child abuse” OR “abuse and neglect” OR “early life stress” OR “child maltreatment” OR “childhood trauma” OR “early life trauma” OR “life change events” OR “psychological trauma”) AND TS=(“clinical trial” OR “interrupted time series analysis” OR “controlled before-after studies” OR quasi-experiment*)
